# Anticancer Activity of Kefir on Glioblastoma Cancer Cell as a New Treatment

**DOI:** 10.1155/2021/8180742

**Published:** 2021-01-12

**Authors:** Arghavan Fatahi, Neda Soleimani, Parviz Afrough

**Affiliations:** ^1^Department of Microbiology and Microbial Biotechnology, Faculty of Life Sciences and Biotechnology, Shahid Beheshti University, Tehran, Iran; ^2^Department of Microbiology and Microbial Biotechnology, Faculty of Life Sciences and Biotechnology, Shahid Beheshti University, Tehran, Iran; ^3^Department of Mycobacteriology & Pulmonary Research, Pasteur Institute of Iran, Tehran, Iran

## Abstract

Kefir drink is one of the most important probiotic products, which is made using kefir microorganisms in fermenting the milk. Numerous investigation have been accomplished in the field of the therapeutic property of probiotic products. In the present study, we assessed the cytotoxic effect of kefir on the rate of growth and increase of glioblastoma cancer cell as the most severe form of brain tumors. In this experimental study, we used a U87 cancer cell line (glioblastoma). The interaction between cancer cells and different concentrations of kefir drink and supernatants at 24 and 48 hours was considered. The cell cytotoxicity of kefir and sedimentation of cell lysate and extract of kefir was assessed using the MTT test after 24 and 48 hours. The result of the MTT test, treatment of the cells with the 48-hour fermented drink, demonstrated the most cell cytotoxicity in comparison with the control group. Results showed that the toxicity effect in all groups was dose-dependent, and by increasing the concentration, cell survival decreased noticeably. The results indicated that the supernatant of fermented kefir drink as a probiotic product has more toxicity and lethality effect on the glioblastoma cancer cell. This product can be utilized as a replacement or a complementary therapy of cancer.

## 1. Introduction

The central nervous system (CNS) and brain tumors are one of the ten cancers leading to death in Iran. Glioma is one of the tumors involving CNS in the result of abnormal divisions of neuroglia cells. According to World Health Organization (WHO) classification, glioma is classified to astrocytoma, oligodendroglioma, and mixed gliomas such as oligoastrocytoma and ependymoma [1]. In 2016, the rate of new suffering in the United States of America was estimated 23770, and among them, 16050 died [2]. Eighty percent of primary malignant brain tumors and 30% of all kind of CNS tumors are caused by glioma [3]. Glioblastoma multiforme, which is the most prevalent and the most acute kind of malignant brain tumors in adults (WHO grade IV), is one of the astrocytic glial cells tumors with histology symptoms such as the increase of mitosis, polymorphisms, endothelial proliferation, and necrosis and disseminates to adjacent tissues rapidly [4]. A side effect of drugs, time-consuming, costly, and inefficiency of this treatment method, explains the obligation of seeking new therapeutic approaches and solutions for the prevention of suffering from cancer. According to mentioned points, one of the considerable replacement solutions of treatment and prevention of malignant brain and spinal cord tumors is utilizing microbial products as kefir. Kefir production is mainly based on the fermentation of milk using starter cultures or kefir grains, which look like small cauliflowers and which contain a complex mixture of lactic acid bacteria, acetic acid bacteria, and yeasts. The complex microbiota in kefir grains has a symbiotic relationship that is responsible for the characteristic taste and flavour of kefir [5]. These products, particularly yoghurt and fermented milk, are also used as vehicles for probiotic bacteria which are reported to have several benefits to the human health [6]. The kefir starter cultures themselves and metabolites of the microorganisms formed during fermentation of kefir lead not only to the formation of lactic acid, acetic acid, ethanol, and carbon dioxide but also to the release of new milk-associated antioxidant micronutrients such as organic acids, amino acids, vitamins (E, B3, B6, and B12), minerals (Se, Fe, Zu, and Mn), and enzymes (glutathione peroxidase, catalase, and superoxide dismutase) [7, 8]. As well as its high nutritional value and excellent taste, kefir and its constituents exhibit a growing number of health-promoting effects including stimulation of the immune system, antihypocholesterolaemic, antihypertensive, antimutagenic, anticarcinogenic, antimicrobial, and antioxidative properties [9]. Probiotics have a substantial role in the treatment and prevention of cancers. The traditional kefir drink is one of the current probiotic products in the world. According to the above-mentioned explanations, we suppose that kefir microorganisms can be efficacious in prevention and treatment of different cancers. Kefir grains have not been used as a therapeutic choice until now; consequently, the aim of this study is utilizing kefir grains and determining its microorganism's effect on glioma cancer cells.

## 2. Material and Methods

### 2.1. Preparation of Kefir Extract

Kefir was supplied from Golestan city. Kefir grains were grown in skim milk at room temperature, and the medium was exchanged daily with new culture medium to maintain grain viability [10]. After the culture process continued for 7 days, the grains can be considered active. Then, 200 cc of fresh skim milk kept at 25°C was added to 20 gr of kefir fungi and incubated at room temperature in two periods of 24 and 48 hours. After 24 hours, kefir fungi were detached, contents were homogenized, and about 5 ml drink was gathered by centrifugation. This process was also repeated after 48 hours. After centrifugation of samples (20 min, 6000 rpm), the remained supernatants were passed from 0.22 *μ*m filter (Millipore) to eliminate the probable contaminations. To investigate the effect of kefir fungi microorganisms on the cells, 4 gr of kefir fungi was soaked in 8 ml PBS buffer and chopped with a sterile scalpel and homogenized with a strip sonicator probe (Vibra-Cell™ VCX130, USA). For breaking the cell wall, kefir fungi soaking in PBS was frozen and defreezed at -80°C for 10 times. Then, sonication was implemented 25 times for 25 sec and rest for 1 min. After that, the supernatant was separated from the sediment with centrifuging (20 min, 6000 rpm). Subsequently, it was incubated at 37° on Nutrient Agar and after 24 h was checked for assuring lack of live cell and contamination. The supernatant and the obtained sedimentation of cell lysate of kefir fungi lysis with sonicator were stored at -20° until the trial time.

### 2.2. Sodium Dodecyl Sulfate-Polyacrylamide Gel Electrophoresis

**Figure 1:**
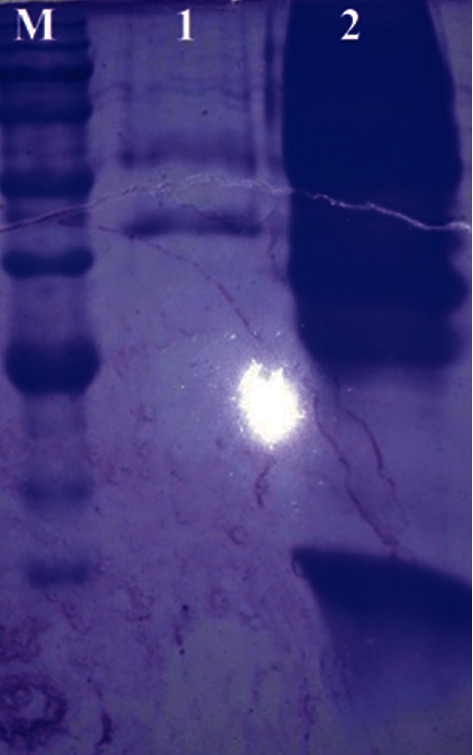
SDS-PAGE of kefir microorganism. M: marker 1. Sedimentation of cell lysate 2. Lysate.

Sodium dodecyl sulfate-polyacrylamide gel electrophoresis (SDS-PAGE) was performed (Bio-Rad) [11], with a 4% stacking gel and a12.5% separating gel. Sonication for 25 min was performed, and then kefir whole-cell lysate and sedimentation of cell lysate were denatured in sample buffer for SDS-PAGE as shown in (Figure 1). Samples were heated in boiling water for 5 minutes and separated electrophoretically at a constant voltage of 90 V. proteins were determined with Coomassie Brilliant Blue R250.

### 2.3. Cell Culture of Cancer Cell Line

U87 glioblastoma cancer cell line (supplied from Cell Bank, Tarbiat Modares University, Tehran) was incubated in DMEM-F12 culture medium with 10% PBS, 100 units penicillin-streptomycin antibiotics at 37°C and 5% CO_2_ [12, 13]. After passaging 3 times, cell count was appropriate for the test.

### 2.4. Cytotoxicity Effect of Kefir on Cancer Cells

Assessment of growth and mortality of cells was accomplished according to the announced protocol. The cells were trypsinized and collected from the flasks and by using hemocytometer slide cell counting was performed. After washing the cells, suspensions of 5 × 103 cells/ml in DMED medium (containing supplements as 2 gr/l of sodium bicarbonate, 2 mM L-glutamine, 100 units/ml penicillin, 100 *μ*g/ml streptomycin, and 10% fetal bovine serum) were seeded in each well of 96-well plates (Nunk) and incubated for 2 h at 37°C in 5% CO_2_. Concentration series of kefir drink, cell lysate, and cell wall of kefir fungi were used to cytotoxicity effect of cancer cell [14, 15]. Plates were incubated at 37°C and 5% CO_2_ for two times 24 and 48 hours. Subsequently, 20 *μ*l of MTT solution was added to each well and incubated at 37°C for an hour to form the formazan crystals. After incubation, the culture medium was offloaded, about 200 *μ*l DMSO solution was added to each well, and then after incubation at room temperature, the optical density (OD) was read at 570 nm wavelength by Absorbance Reader (ELx800™, BioTek, USA) [11].

### 2.5. Statistical Analysis

The software SPSS, version 22.0 (IBM Corp., NY, USA), was used for statistical analysis. Results were reported with one-way analysis of variance ANOVA and the standard error described as mean ± SEM for all the data. A *P* value < 0.05 was considered statistically significant.

## 3. Results

### 3.1. Result of Sodium Dodecyl Organism Gel Electrophoresis

The result of sodium dodecyl sulfate-polyacrylamide gel electrophoresis showed different bounds in gel.

### 3.2. Cytotoxicity Assay

The MTT test finding demonstrated the effect of the treatment on growth and mortality of the cells by turning the yellow color of tetrazolium solution to purple which is in the result of formazan crystal formation by mitochondrial succinate dehydrogenase enzyme activity. Subsequently, purple color is observed when the cell is alive, and by comparison of the color intensity in treated wells and control wells, increase or decrease of cell count will be revealed. The rate of cell viability under treatment influence is as follows: (%) Cell viability = (average of optimal density of treatment)/(average of optimal density of control) × 100. U87 cells were treated with two samples of kefir drink which were equal in all of the preparation processes except the fermentation duration so as to consider the effect of fermentation duration in drink function. The rate of cell growth assessment demonstrates that in treatment with sample A drink (drink supernatant with 24 hours fermenting duration), by increasing the concentration, cell viability decreases. At a 24-hour period, in 15 mg/ml concentration, we observed the least cell viability (37%), and in 1.5 mg/ml concentration, only a 17% decrease in cell growth was observed. At a 48-hour period, we observed the same finding as described above (the most cell viability: 90% in 1.5 mg/ml concentration and 44% in 20 mg/ml concentration).

**Figure 2:**
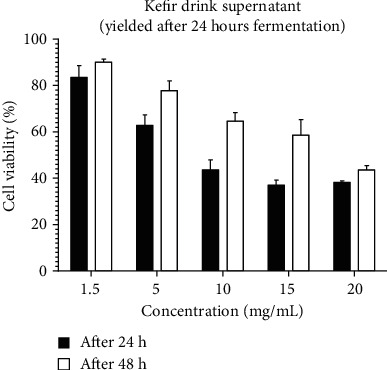
Effect of kefir drink supernatant (yielded after 24 hours fermentation) on U87 cell viability after 24 and 48 hours (*P* value ≤ 0.5).

**Figure 3:**
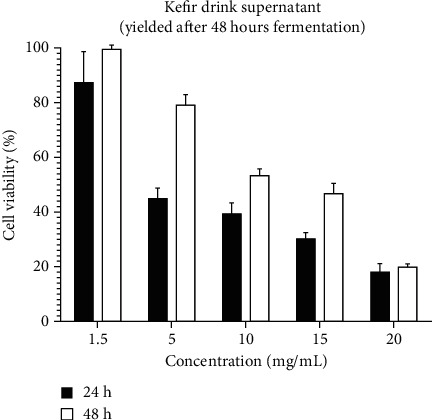
Effect of kefir drink supernatant (yielded after 48 hours fermentation) on U87 cell viability after 24 and 48 hours (*P* value ≤ 0.5).

In Figures 2 and 3, treatment of the cells with the second sample of drink, with 48-hour duration of fermentation, showed similar effects on the cells. In both of the studies at 24 and 48 hours, the most and the least cell viability was observed, respectively, in 1.5 and 20 concentrations. Comparison of the MTT test results showed that, similarly in both of the treatments, the cytotoxicity of the drinks has reduced at a 48-hour period in comparison with the 24-hour period. Likewise, the comparison of two treatments at 24-hour period demonstrates that all the concentrations except 1.5 mg/ml demonstrated that the sample of kefir drink which was produced during 24 hours fermenting kefir drink supernatant (yielded after 24 hours fermentation) has more cytotoxicity effect in comparison with kefir drink supernatant (yielded after 48 hours fermentation). At 48 studies, this process was observed except for 1.5 and 5 mg/ml concentrations.

**Figure 4:**
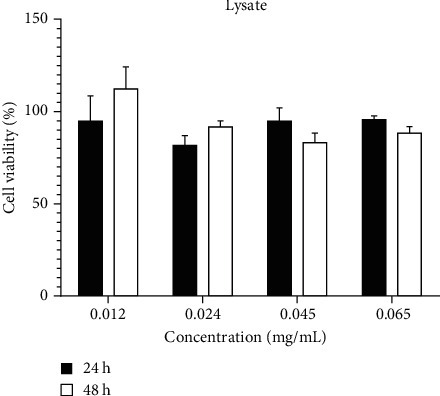
Effect of lysate of microorganism of kefir on U87 cell viability after 24 and 48 hours (*P* value ≤ 0.5).

In the investigation of homogenized kefir, fungi extract supernatant effect on, no changing in the process was observed. The most rate of cell death after treatment at the 24-hour period in 0.024 mg/ml concentration decreased 19% and during 48 hours in 0.045 mg/ml concentration decreased 17%. The impressive point was lack of death and the effect of the sample on growth that increased by 12% in 0.012 mg/ml concentration (Figure 4).

**Figure 5:**
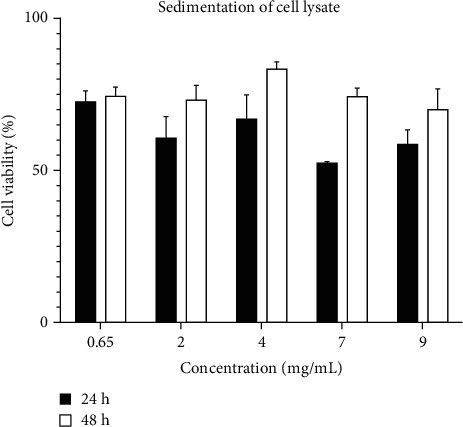
Effect sedimentation of cell lysate on U87 cell viability after 24 and 48 hours (*P* value ≤ 0.5).

In the result of treatment with kefir fungi sedimentation of cell lysate (Figure 5), in the 24-hour test, the most viability was observed in 0.65 mg/ml (72%) concentration and the least viability was observed in 7 mg/ml concentration (IC_50_). In the 48-hour test, the rate of cell viability in 9 mg/ml and 4 mg/ml concentrations was 69% and 83%, respectively. The obtained results of the supernatant and the sedimentation of cell lysate assessment showed that the most rate of the cell death after treatment with bacterial supernatant at 24 hours in 0.024 mg/ml concentration and at 48 hours 0.045 mg/ml concentration showed 19% and 17% growth reduction, respectively. This issue demonstrates that some of the microbial metabolites in this process can lead to the death of glioblastoma cancer cells line. In the result of treatment with kefir fungi sedimentation of cell lysate, at 24-hour test, the most viability rate and the least viability rate were observed in 0.65 mg/ml and 7 mg/ml concentrations, respectively (IC_50_). At 24-hour test, cell viability in 9 mg/ml concentration was 69%, and in 4 mg/ml, concentration was 83%. These results show that the microbial metabolite can result in death of glioblastoma cancer cells line.

## 4. Discussion

Microbial metabolites have an essential and undeniable role in the treatment of disease including cancers. Nowadays, there are plenty of new therapeutic procedures for glioma treatment under investigation. Kefir traditional drink is one of the current probiotic drinks in the dairy industry in the world. This probiotic drink has attracted researchers' attention as an option for the treatment of numerous diseases in recent years. Some of the kefir properties, such as antimicrobial use wound healing, digestive system immunity augment, regulation of cholesterol absorption, and antitumor and antimetastatic effects on various cancer cell lines, are just some of the therapeutic properties of this probiotic foodstuff [16]. Probiotics and the produced metabolites including organic acids, bacteriocins, and peptides can interfere in metabolite pathways such as proliferation regulation, cell distinction, apoptosis, metastasis, and angiogenesis. *Lactobacillus acidophilus* and *L. delbrueckii* bacterial supernatants revealed their anticancer property on AGS MCS-7 SW620 and HT-29 cancer cell lines by the induction of the caspase-3-dependent apoptosis pathway and reduction of Bcl2 expression. *Saccharomyces cerevisiae* yeast has also demonstrated significant antitumor effects and growth inhibition in HepG2 cancer cells and breast cancer. Kefir is likewise a foodstuff including a collection of probiotic microorganisms that the majority of these probiotics are lactic acid bacteria (LAB). In this study, observations showed that supernatant without kefir cells (A and B samples) significantly inhibits the cell proliferation in U87 cell line in a dose-dependent manner (Figure 5). KG-1 blood cancer (acute erythroleukemia) cell line treatment with kefir extraction without cell demonstrated the induction of apoptosis and necrosis and also decrease of the erythroleukemia cancer cell line proliferation [17]. An infected mouse with 4TI breast cancer cell line with a kefir drink diet during 28 days demonstrated a significant decrease in weight and size of tumors and increase of T helper and cytotoxic cells production along with the antimetastatic effect of kefir on tumors [18]. Kefir extraction causes the decrease of TGF-*α* gene expression in HuT-102 line of T-lymphocyte cancer cells (HTLV-1-positive malignant T-lymphocyte) and consequently decreases 98% of cell proliferation in treatment with 60 mg/ml during 24 hours [19]. In addition, extraction of microorganisms in homogenized kefir and its sedimentation of cell lysate was surveyed, and the findings demonstrated that the produced cell lysate which had a significant viscosity and jelly-like appearance, in applied concentrations, showed a little toxicity. Khoury et al.'s findings showed that gastric cancer cells line SGC 7901 treatment with kefir causes a dose-dependent decrease in cell proliferation [20]. Moreover, in another study, blood cancer KG-1 cell line (acute erythroleukemia) treatment with kefir extraction without cell causes a decrease of erythroleukemia cancer cell line proliferation [17]. Intestinal Caco-2 and HT-29 cell line treatment with kefir extraction demonstrated time- and dose-dependent inhibition of the cell proliferation [19]. They also suggested that probiotic yoghurt might be a promising product for cancer management. The results in this study are also in accordance with the findings of [19] who confirmed the inverse relationship between kefir and fermented dairy (containing live probiotics) consumption and the incidence of cancer [21]. Due to the properties of this drink, kefir can be of interest to the food industry, especially the dairy, for the production of this valuable product.

## 5. Conclusion

Kefir can be important in the dairy industry. The antitumor effects of this drink can have a special place in the diet. The present study results showed that kefir significantly reduced the growth rate of the U87 cell line. Consequently, kefir can be an option for a glioblastoma brain tumor which is the most acute type of glioma, and the rate of the suffering of patients is still low. Therefore, a more accurate survey of kefir function mechanism and its effectiveness, inducing apoptosis, are considered in future investigations.

## Data Availability

The data used to support the findings of this study are available from the corresponding author upon request.
